# Searching for Atherosclerosis Biomarkers by Proteomics: A Focus on Lesion Pathogenesis and Vulnerability

**DOI:** 10.3390/ijms242015175

**Published:** 2023-10-14

**Authors:** Gabriele Nieddu, Marilena Formato, Antonio Junior Lepedda

**Affiliations:** Department of Biomedical Sciences, University of Sassari, 07100 Sassari, Italy; ganieddu@uniss.it (G.N.); formato@uniss.it (M.F.); Antonio Junior Lepedda (A.J.L.)

**Keywords:** proteomics of the atherosclerotic plaque, lipoproteomics, plaque vulnerability, atherogenesis

## Abstract

Plaque rupture and thrombosis are the most important clinical complications in the pathogenesis of stroke, coronary arteries, and peripheral vascular diseases. The identification of early biomarkers of plaque presence and susceptibility to ulceration could be of primary importance in preventing such life-threatening events. With the improvement of proteomic tools, large-scale technologies have been proven valuable in attempting to unravel pathways of atherosclerotic degeneration and identifying new circulating markers to be utilized either as early diagnostic traits or as targets for new drug therapies. To address these issues, different matrices of human origin, such as vascular cells, arterial tissues, plasma, and urine, have been investigated. Besides, proteomics was also applied to experimental atherosclerosis in order to unveil significant insights into the mechanisms influencing atherogenesis. This narrative review provides an overview of the last twenty years of omics applications to the study of atherogenesis and lesion vulnerability, with particular emphasis on lipoproteomics and vascular tissue proteomics. Major issues of tissue analyses, such as plaque complexity, sampling, availability, choice of proper controls, and lipoproteins purification, will be raised, and future directions will be addressed.

## 1. Introduction

**Figure 1:**
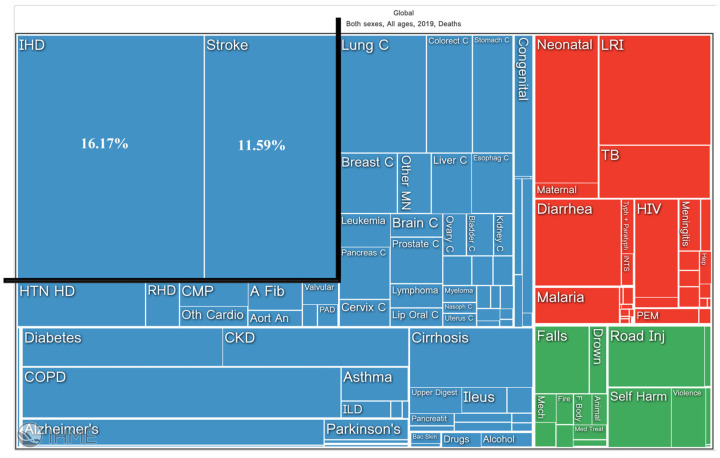
Picture showing the contribution of major acute cardiovascular events such as IHD and stroke to global deaths in 2019 [10]. Blue: non-communicable diseases. Red: communicable, maternal, neonatal, and nutritional diseases. Green: injuries.

Atherosclerosis stands as the primary contributor to the prevalence of cardiovascular disease (CVD), the leading cause of death and illness in developed nations, being responsible for major acute clinical events, such as ischemic heart disease (IHD), and stroke (16.17% and 11.59% of global deaths in 2019, respectively) (Figure 1).

It is a multifactorial disease, in which several genetic (e.g., high LDL-cholesterol, low HDL-cholesterol, hypertension, diabetes, gender) and environmental (e.g., high-fat diet, smoking, lack of exercise) factors play a role. Atherogenesis begins with the formation of a fatty streak into the subendothelial space of medium and large arteries in the first decade of life [1], which may evolve toward the development of an atherosclerotic plaque with significant intra- and inter-individual heterogeneity in terms of both growth rate and pathologic features [2]. The occurrence of plaque rupture or erosion leading to atherothrombosis is the underlying pathology responsible for major acute events, such as stroke, acute coronary syndrome (ACS), and peripheral artery occlusion [3,4]. In this respect, several lines of evidence suggest that unstable plaques are characterized by a more pronounced inflammation and proteolysis [5,6], as well as a pro-oxidant environment [7,8]. To date, considerable efforts have been focused on both elucidating the determinants of carotid plaque vulnerability and identifying reliable and specific markers for the susceptibility of plaques to ulceration, in order to prevent significant adverse clinical events associated with thrombosis and artery occlusion [9].

**Figure 2:**
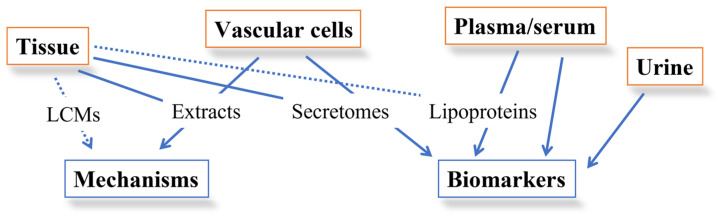
Overview of the main targets of proteomic studies searching for both mechanisms of atherogenesis and biomarkers of atherosclerotic lesion presence and progression. Dotted lines represent partially unexplored paths. LCMs, laser-captured microdissections. Modified from [43].

Gaining a thorough understanding of the arterial protein networks involved in the development of plaque formation, as well as their subsequent alterations during the early stages of atherosclerosis, may contribute to implementing personalized therapies [11]. Accordingly, over the past two decades, advancements in proteomic tools have enabled the application of large-scale technologies to unravel pathways involved in atherosclerotic degeneration, also allowing the identification of new circulating markers that are useful as early diagnostic traits or as targets for new drug therapies, besides being indicative of physiological alterations associated with vascular aging [12]. To tackle this challenge, several proteomic approaches, such as one-dimensional and two-dimensional electrophoresis (1-DE and 2-DE) followed by mass spectrometry (MS) analyses, protein arrays [13,14], and gel-free MS-based proteomics, have been applied to different matrices of human origin [15], such as vascular cells [16,17,18] and arterial tissues, targeting total extracts [19], extracts from laser capture microdissection (LCM) [20], secretomes [21], plasma/serum [22], urine [23], and purified plasma lipoprotein fractions [24,25] (Figure 2). In addition, platelets, which are known to be key modulators of inflammation and immune response [26,27], as well as the primary target for the prevention of arterial thrombus formation, have been investigated through different -omics approaches including proteomics, lipidomics, metabolomics, transcriptomics, and translatomics, as recently described by Gutmann et al. [28]. Besides this, proteomics has been applied to animal models such as apo E knock-out mice [29,30,31,32], rabbits [33,34,35], and pigs [36,37] to unveil significant insights into the molecular mechanisms influencing atherogenesis. Although in its infancy, further support in the search for additional markers of atherogenesis and plaque degeneration has been provided by lipidomics of plasma/lipoprotein fractions [38,39,40], as well as of atherosclerotic tissues [41,42].

Herein, the results of an in-depth literature search using MEDLINE (PubMed) were reported, with the aim to provide an overview of the last 20 years of proteomics and lipidomics studies on atherosclerosis, with particular emphasis on lipoproteins and atherosclerotic tissues.

## 2. Application of MS-Based Technologies to the Study of Lipoproteins Involvement in Atherogenesis

Lipoproteins are supramolecular complexes that deliver insoluble lipids from the tissues where they are synthesized to those that metabolize or store them. They consist of a hydrophobic core, mainly composed by triacylglycerols and cholesteryl esters, stabilized by a coat of amphipathic compounds, namely phospholipids, unesterified cholesterol, and proteins, with the latter referred to as apolipoproteins (apo) [44].

Lipoprotein classes that have been described so far differ in terms of chemical compositions, physical properties, as well as metabolic functions, and may be classified according to their densities in chylomicrons (d < 0.95 g/mL), very low-density lipoproteins (VLDL, d < 1.006 g/mL), low-density lipoproteins (LDL, 1.019 < d < 1.063 g/mL) and high-density lipoproteins (HDL, 1.063 < d < 1.21 g/mL) [45]. A further class of lipoprotein particles is known as lipoprotein(a) or Lp(a), an LDL-like particle that carries a heavily glycosylated multi-kringle protein named apolipoprotein(a), linked to apo B100 by a single disulfide bond. The physiological role of Lp(a) is still unknown, although it has been demonstrated to be a risk factor for cardiovascular disease [46]. Lipoproteins, especially low-density and high-density lipoproteins (LDL and HDL), have attracted a great deal of interest because of their implications in the development of cardiovascular diseases. LDL is the main carrier of cholesterol to the peripheral tissues, and a well-established major risk factor for atherosclerosis and cardiovascular diseases [47]. Indeed, in 1995, Williams and Tabas published the “response-to-retention” hypothesis; according to this theory, early events in atherogenesis are related to the selective retention of LDL in the sub-endothelial space of the artery wall by means of specific interactions with some extracellular matrix components [48]. HDL exerts significant vascular protective effects by facilitating the elimination of cholesterol excess from cells and its transport to the liver (reverse cholesterol transport) [49], besides performing antioxidant, anti-inflammatory, and anti-thrombotic activities [50]. Furthermore, Vaisar et al. reported that HDL is enriched in several proteins involved in the complement cascade, suggesting a role in innate immunity [51].

### 2.1. Lipoproteomics

**Figure 3:**
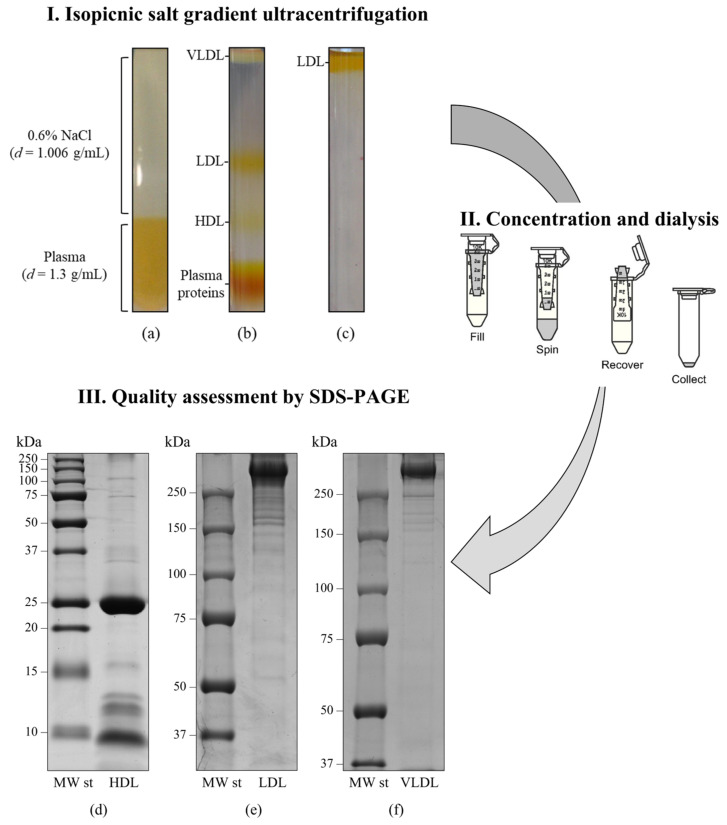
Lipoprotein purification and quality assessment procedures workflow. (**I**). Isopycnic salt gradient ultracentrifugation. (**a**) Ultracentrifugation tube containing 0.9 mL of plasma sample combined with NaBr, overlaid with 2.1 mL of a 0.6% NaCl solution; (**b**) self-generated density gradient following ultracentrifugation at 541,000× *g* for 3 h at 4 °C in a TL-100 series ultracentrifuge equipped with a TLA-100 fixed-angle rotor (Beckman Coulter, Brea, CA, USA), showing the three main lipoprotein classes; (**c**) LDL on the top of the tube following a further flotation step at d = 1.063 g/mL. (**II**). Concentration and dialysis using Amicon Ultra-0.5 mL centrifugal filter units (10 KDa MWCO, Merck-Millipore, Darmstadt, Germany). (**III**). Quality assessment using sodium dodecyl sulfate—polyacrylamide gel electrophoresis (SDS-PAGE). Representative mono-dimensional profiles of HDL (**d**), LDL (**e**), and VLDL (**f**) fractions obtained by SDS-PAGE in either 12% T (for HDL, under reducing conditions) or 6% T (for both LDL and VLDL, under non-reducing conditions) resolving gels (modified from Nieddu et al. [55]).

In recent years, it has become more evident that the protein composition of each lipoprotein class is largely responsible for its various functions, particularly in relation to pathological conditions, including atherosclerosis. Consequently, acquiring detailed information about the composition and structure of their apolipoproteins cargo could potentially shed light on their involvement in atherogenesis and on the progression of atherosclerotic lesions, ultimately leading to the formation of vulnerable plaques. In this respect, the method adopted for the isolation of lipoproteins has to be taken into great consideration, as it may significantly affect the protein content of the purified particles [52]. Traditional methods, established in the 1950s [53], include ultracentrifugation in high-salt media containing KBr or NaBr (Figure 3). It is worth mentioning the recent review by Chary and Hedayati, in which some techniques used to isolate and measure LDL and HDL subclasses are reported, including agarose gel electrophoresis, nuclear magnetic resonance spectroscopy (NMR), high-performance liquid chromatography (HPLC), and LipoPrint [54].

Several lipoproteomic studies have been published using these procedures of lipoprotein isolation (see Supplementary Materials 1 of Finamore et al. [56]). However, the high ionic strength and the high centrifugal field forces might cause either the dissociation of proteins or their exchange between different lipoprotein classes, potentially altering the pattern of associated exchangeable apolipoproteins. Indeed, some of these studies reported a loss of proteins after a second step of ultracentrifugation [57,58,59]. Some others employed two ultracentrifuge procedures, using both salts and other compounds, such as sucrose and iodixanol [60,61], reporting comparable results. In this respect, Stahlman et al. reported that deuterium oxide (D_2_O) has to be preferred over salts for isolating both LDL and HDL, due to the abovementioned high ionic strength of the isopycnic medium [62]. Alternatively, lipoproteins can be isolated by means of immunopurification methods that rely on specific antibodies for the dominant protein of each class. Although this procedure does not lead to the loss of weakly associated proteins, it may co-purify nonspecifically associated proteins, such as serum contaminants and/or other lipoprotein fractions carrying the same antibody target (e.g., apolipoprotein A-I is the main HDL apolipoprotein, but it is also present in both VLDL and LDL fractions). Other lipoprotein isolation methods that have been applied in lipoproteomic studies involve electrophoretic techniques, specifically free solution isotachophoresis [63], and chromatographic techniques, such as fast protein liquid chromatography [64], and size exclusion/affinity chromatography [65,66]. Besides the method used for lipoprotein purification, the performance of the mass spectrometry analysis as well as the sample heterogeneity are major contributors to the differences reported from study to study (see Davidson et al. 2022 [67] for a detailed discussion).

An updated list of all identified HDL (last update 20 April 2023) and LDL (last update 18 February 2015) proteins, ranked according to the frequency of identification in MS studies, can be downloaded at the “The Davidson/Shah lab” website (www.DavidsonLab.com, accessed on 14 July 2023).

According to “The HDL Proteome Watch Database” by the Davidson Laboratory, which has included 51 proteomics studies on HDL, published up to 2022, 1030 proteins have been identified, of which 285 (defined as “likely” HDL proteins) have been reported by at least three different laboratories. Gene ontology analyses on the “likely” HDL proteins revealed that they are implicated in several biological processes including lipid transport, hemostasis/protease inhibition, inflammation/acute phase, immunity/anti-microbial and cell/heparin binding [67]. With regard to LDL, the Davidson database lists 60 proteins from four proteomics studies up to 2015; amidst these, 22 (defined as “likely” LDL proteins) were identified independently by at least two studies (apolipoproteins A-I, A-II, A-IV, B, C-I, C-II, C-III, C-IV, D, E, F, J, L1, M, (a), serum amyloid A1, A2, A4, albumin, alpha-1-antitrypsin, cathelicidin antimicrobial peptide, dermcidin, fibrinogen alpha chain).

**Figure 4:**
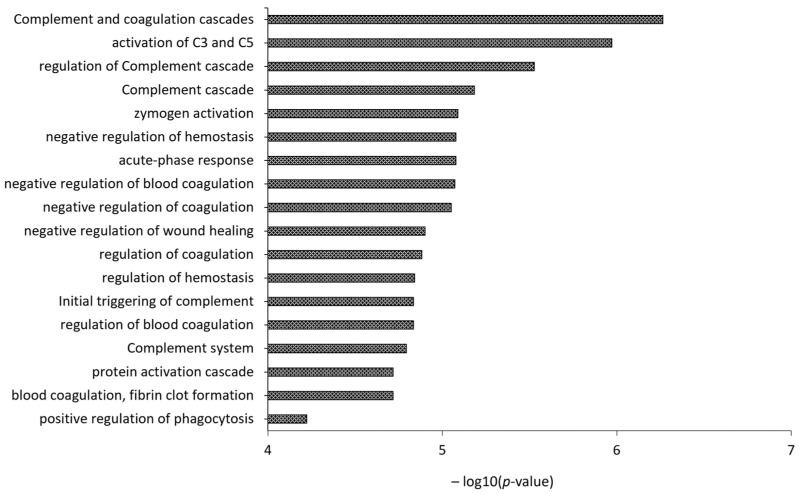
Gene Ontology (GO) analysis of the 21 proteins identified by Finamore et al. [56], not yet included in the “likely” LDL proteins list of the Davidson’s Lab database, but already reported to be associated with LDL by either Dashty et al. [68] or Bancells et al. [69].

Recently, by applying shotgun proteomics to identify the apolipoprotein signatures of both HDL and LDL from healthy volunteers and atherosclerotic patients with different carotid plaque typologies, we enriched the list of LDL-associated proteins with 21 proteins that, by means of gene ontology analysis, were shown to be involved in complement and coagulation cascades, and the acute-phase response, potentially doubling the protein species of LDL cargo [56] (Figure 4).

Only a few studies dealt with the VLDL proteome, mostly comparing it with that of LDL and/or HDL under physiological conditions [57,61,62,70,71,72,73]. Among them, Dashty et al. identified 95 VLDL- and 51 LDL-associated proteins (of which 39 shared proteins and 56 and 12 were unique for VLDL and LDL, respectively), including all known apolipoproteins and lipid transport proteins, and a set of coagulation proteins, complement system proteins, and anti-microbial proteins [68].

Scant data have been reported on the Lp(a) proteome so far. Von Zychlinski et al. performed two-dimensional liquid chromatography-coupled tandem mass spectrometry on highly purified Lp(a) particles that underwent tryptic digestion, identifying with high confidence 35 proteins involved in two major biological processes of lipid metabolism and response to wounding, the latter including the processes of coagulation, complement activation, and inflammatory response [74]. Bourgeois and colleagues performed a discovery analysis on both Lp(a) and LDL identifying 154 associated proteins, whereas a differential analysis between the Lp(a) and LDL proteomes showed 15 proteins preferentially associated with Lp(a) with potential implications for acute inflammatory response, extracellular structure organization, and protease inhibition [75]. Very recently, Mueller et al. set up a method for the isolation of Lp(a), starting from very small volumes of plasma samples (0.4 mL), suitable for different applications including in-depth analysis by mass spectrometry, opening up the opportunity to perform proteomics on casuistries with a limited availability of samples [76].

Over the past two decades, numerous proteomic studies have been conducted on purified lipoprotein fractions, investigating both physiological and pathological conditions, the latter including coronary artery disease (CAD), acute myocardial infarction (AMI), chronic kidney disease (CKD), end-stage renal disease, type 1 and 2 diabetes mellitus (T1DM and T2DM), and experimental atherosclerosis (Table 1). 

To the best of our knowledge, the application of both proteomics and lipidomics to lipoproteins from atherosclerotic tissue remains a path that is partially unexplored, probably because of the intrinsic issues in purifying them from the extracellular lipid-rich matrix of the plaque.

### 2.2. Lipidomics

Despite providing valuable insights into the various functions of each lipoprotein class in relation to cardiovascular disease, there is currently a lack of the additional information required to assess the risk of acute clinical event onset. Indeed, the multifaceted biological functions of lipoproteins, especially HDL, arise from the combined protein and lipid components, and any disruptions or modifications of these components can lead to dysfunctional particles [122]. In recent years, there has been a growing interest in plasma lipidomics due to compelling evidence linking specific plasma lipid species to the development of atherosclerosis [38,123,124,125,126,127,128,129], and the onset of adverse clinical events [39,130,131,132,133,134]. However, to date, only a limited number of studies have explored the association between biologically active lipids, particularly those associated with their lipoprotein carriers, and cardiovascular disease (CVD), as also recently reviewed by Ding and Rexrode [135].

The majority of the published studies have evidenced changes in the HDL phospho- and sphingo-lipidomes in relation with type 1 diabetes [136], type 2 diabetes [137,138], obesity and metabolic syndrome [139,140], dyslipidemia [141,142], and experimental atherosclerosis [143,144]. Furthermore, it has been shown that either Pitavastatin treatment [145] or lifestyle changes, involving weight loss and physical activity [146], were able to revert the HDL lipidome in metabolic syndrome. Besides this, both statin treatment [147] and phytosterol and omega-3 diet supplementation [148] were found to induce changes in LDL lipidome, resulting in a decreased cardiovascular risk.

The lipidome profiles of both HDL and LDL were also examined in relation to CAD and ACS [149,150,151], showing distinct profiles with respect to healthy controls, whereas the HDL phosphosphingolipidome was shown to have distinct signatures in subjects affected by hyperalphalipoproteinemia, a rare condition characterized by high plasma HDL-cholesterol levels associated with an increased risk of early CAD onset [152,153].

From our side, in order to identify further markers of atherosclerotic plaque vulnerability, we performed lipidomics via selected reaction monitoring-based high-performance liquid chromatography–tandem mass spectrometry (SRM-based HPLC-MS/MS) analysis on plasma HDL, LDL, and VLDL fractions purified from twenty-eight patients with advanced carotid lesions, sorted according to the typology of plaque by ultrasonography into hypoechoic, or “soft”, and hyperechoic, or “hard”. By this approach, we evidenced a significant dysregulation of LDL phosphatidylethanolamine (38:6), sphingomyelin (32:1), and sphingomyelin (32:2) between the two groups of patients, in line with the current knowledge of the key roles of these phospholipids in lipoprotein metabolism and cardiovascular disease [55].

With the aim of obtaining a deeper insight into lipoproteins’ metabolism in association with CVD, very recently, Wang and coworkers developed a high-resolution method to depict both the proteome and lipidome of lipoproteins, and their interconnections [40]. Using this approach, they demonstrated that the combined results yielded enhanced differentiation between ACS and healthy individuals compared to either proteomics and/or lipidomics alone.

## 3. Atherosclerotic Plaque Dissection through Proteomics

Although tissue analyses frequently provide useful information, there are major issues in analyzing human atheromas specimens. In fact, the atherosclerotic plaque environment is a very complex tissue in terms of cell types involved, as well as inflammatory, proteolytic, and oxidative mechanisms, which ultimately reflect a systemic condition resulting from the long-term persistence of multiple environmental and genetic risk factors. In this respect, besides vascular smooth muscle cells and endothelial cells, plaque contains many types of inflammatory cells, filtered plasma proteins, newly formed extracellular matrices, cellular debris and end-products of lipid and protein oxidation [2,3,4]. This issue could be partially overcome by selecting specific cells or plaque areas using LCM, a path explored only to a limited extent by proteomics, which holds significant promises. Another critical point of the in situ analyses is the choice of an appropriate control. To minimize inherent tissue differences, it would be desirable to use control specimens from the same vascular district of the same patient. Furthermore, specimens should be taken from surgical endarterectomy rather than from post-mortem material, to avoid tissue degradation before the analysis. The availability of a poor number of human specimens could be a limitation, as well. Because of the heterogeneity of the different types of advanced lesions in terms of fibrous cap thickness, inflammatory and proteolytic components, calcifications, surface erosion, thrombosis, and intraplaque hemorrhage, a careful histochemical classification prior to biochemical analyses is desirable [154,155].

In the last few years, different proteomic technologies have been applied to diseased human tissues to provide further insights into the molecular mechanisms of advanced atherosclerotic plaque development, as well as to identify diagnostic traits of plaque instability useful as therapeutic targets or as markers for patient follow-up. With this aim, both plaque extracts and secretomes, obtained by culturing different typologies of plaque segments, have been the topic of these studies. Besides this, to delve deeper into the mechanisms underlying the early stages of lesion formation, some animal models, mainly rodents, have been investigated.

### 3.1. Studies on Human Plaque Extracts

Since 2003, about twenty studies on human atherosclerotic plaque proteomics have been published (Table 2). Most of them focus on coronary or carotid atherosclerosis, using mostly 1-DE, 2-DE, or two-dimensional difference gel electrophoresis (2D-DIGE) coupled with image analysis to reveal differentially expressed proteins, followed by protein identification by Matrix Assisted Laser Desorption Ionization–Time of Flight (MALDI-TOF) MS or liquid chromatography (LC) MS/MS. Some limitations of these studies include the low number of analyzed specimens and the choice of the appropriate controls, usually from unaffected vascular districts and/or patients, thus omitting intrinsic tissue and/or inter-individual differences. Sometimes, a proper validation undertaken on an independent cohort of subjects by complementary approaches is also missing.

The first large-scale protein profile of human atherosclerotic coronary arteries was obtained by Bagnato et al., who were able to identify 806 unique proteins with high confidence [159]. They applied the direct tissue proteomic (DTP) approach, a method developed by Hwang et al. for the identification of candidate markers from formalin-fixed paraffin-embedded cancer specimens [174], to paraffin or frozen blocks from 35 coronary atherosclerotic lesions, classified by histopathological examination in early, intermediate, and advanced stages. In particular, different plaque areas were laser-microdissected from both paraffin and frozen section specimens and subjected to tryptic digestion followed by LC MS/MS to obtain area-specific proteomic information. Frozen sections were also homogenized; the obtained proteins were resolved by SDS-PAGE, and then analyzed by LC MS/MS. Moreover, they used AQUA (absolute quantitation) methodology to quantify Stromal Cell-derived Factor 1 α (SDF1-α) and growth factors not detected by the above-mentioned methods. By this approach, Bagnato et al. showed that LCMs applied to plaque proteomics represent a suitable tool to compare different areas of the plaque, such as necrotic core and shoulders/fibrous cap, providing valuable spatial information.

LCM was also applied by de la Cuesta et al. to isolate the intimal layers from human atherosclerotic coronaries, and from pre-atherosclerotic coronary and radial arteries, which were analyzed by 2D-DIGE coupled to MALDI-TOF/TOF MS protein identification [163]. They evidenced an altered expression of 13 proteins involved in key processes of plaque development, including vascular smooth muscle cells (VSMCs) migration, extracellular matrix (ECM) composition, coagulation, apoptosis, heat shock response, and intraplaque hemorrhage deposition.

With the aim of going deeper into the plaque proteome, Hao et al. applied multidimensional LC-MS/MS to the analysis of two extract pools from thirty-eight carotid plaques. In total, 4702 proteins were identified, including many low-abundance proteins previously unidentified by MS [165]. In this proof of concept study, an impressive number of proteins was identified, most of them being associated with pathways related to atherogenesis and plaque progression, showing the great potential of proteomics applied to atherosclerosis.

Alonso-Orgaz et al. performed the first proteomic analysis on thrombus, recovered from patients with ST-segment elevation acute myocardial infarction (STEMI) by percutaneous intracoronary thrombectomy during primary angioplasty. By applying three different proteomic approaches, they were able to identify a total of 708 proteins in the thrombus, including the protein death-inducer obliterator 1, which was proven to be increased in the plasma of STEMI patients, paving the way in the search for biomarkers of thrombosis [167].

By applying proteomics to different regions of human carotid atherosclerotic arteries, namely, internal control, fatty streak, plaque shoulder, plaque center, and fibrous cap, Yuan’s team was able to identify site-specific alterations, some of them gender-specific, with clear associations to extracellular matrix remodeling processes. Overall, their results suggest that women develop plaques with a lower inflammatory profile and a greater stability than men [168,172].

With the aim of identifying novel markers of plaque disruption, Lee et al. performed proteomics on plaque debris obtained during coronary angioplasty, recovered using filterwire devices, identifying 423 proteins. Patients who underwent angiography without stenting were used as controls [169]. Matrix metalloproteinase-9 (MMP-9) was found to be significantly increased in lipid-rich plaques, as well as in the plasma of patients following plaque disruption by stenting. The developed method represents a novel approach to the discovery of circulating biomarkers of plaque erosion, which surely represents one of the most important pathological mechanisms underlying major acute clinical events.

Atherogenesis is a degenerative disease of arteries that remains silent for decades, although early lesions can appear in children or young adults. Identifying arterial protein networks and their alterations during the early stages of atherosclerosis may have great diagnostic and therapeutic potential, as well as being useful for acute clinical event prevention. In this respect, Herrington et al. performed a study focused on identifying features of early atherosclerosis by analyzing one hundred coronary and one hundred aortic autoptic specimens from young adults. By this approach, they were able to identify 1925 proteins, as well as numerous networks and pathways associated with early atherosclerosis. Furthermore, based on the obtained results, they selected a panel of thirteen plasma proteins that were demonstrated to be effective in predicting the presence of coronary artery disease in an independent clinical cohort of patients [11].

### 3.2. Studies on Human Plaque Secretomes

Theoretically, the secretome should reflect the tissue-specific metabolic activities under both physiological and pathological conditions. With respect to plasma, it is also characterized by a lower number of protein species as well as a narrower dynamic range of concentrations. Therefore, it may represent a rich source of biomarkers with potential implications in clinical practice.

The approach of studying the cultured plaque secretome was pioneered by the teams of Vivanco and Egido, and was adopted successfully in some research in the following years (Table 3). To obtain the secretome (the whole set of proteins released/secreted by the plaque), specimens are cultured in serum-free medium, and the supernatant is collected for proteomics analysis. The method set up by Duran et al. analyzed the secretomes from different regions of the same carotid specimens obtained by endarterectomy (normal segments, non-complicated plaques, and complicated plaques with a thrombus), thus avoiding the intra- and inter-individual variability of the control specimens, through 2DE coupled with MS [175].

This method was applied by Martin-Ventura et al. to the analysis of the protein secretion profiles obtained from 35 cultured atherosclerotic plaques (10 femoral, 25 carotids) and 36 control arteries (24 mammary, 12 radial) with the aim of identifying new biological markers potentially released by the arterial wall into the plasma [176]. They showed that heat shock protein 27 (HSP27) secretion into the culture medium was significantly lower in atherosclerotic plaques and barely detectable in complicated plaque supernatants compared to control arteries, as confirmed by western blotting analysis. They also proposed HSP27 as a possible marker of atherosclerosis, since they evidenced a 20-fold reduction in its plasma levels in patients with carotid stenosis with respect to healthy controls. Interestingly, by analyzing the 2DE protein profiles of extracts from nineteen stable and twenty-nine unstable human carotid plaques, we found a reduced expression of HSP27 in the latter [6]. The abovementioned teams evaluated the effects of incubation with atorvastatin, a 3-hydroxy-3-methylglutaryl CoenzymeA reductase inhibitor, on the secretomes of cultured atherosclerotic plaques [177]. They identified 24 proteins that were increased and 20 proteins that were decreased in atherosclerotic plaque supernatants compared to controls. It is noteworthy that the presence of atorvastatin in the culture medium reverted the secretion of 66% of proteins to their control values. Some of these proteins may contribute to plaque instability, representing potential pharmacological targets.

By applying surface-enhanced laser desorption/ionization (SELDI) TOF MS profiling to secretomes from seven carotid atherosclerotic plaques and seven mammary artery specimens, Blanco-Colio et al. identified soluble tumor necrosis factor-like weak inducer of apoptosis (sTWEAK), which was less expressed in plaques with respect to normal arteries. Notably, this protein was found negatively associated with carotid intima-media thickness, an index of subclinical atherosclerosis, in a group of 106 asymptomatic subjects enrolled for a general health check-up [178].

De la Cuesta et al. performed LC MS/MS analyses on secretomes from human atherosclerotic coronary arteries, pre-atherosclerotic coronaries, and mammary arteries, identifying 64 proteins, 15 of which were not previously reported in plasma. They showed that, due to the reduced dynamic range of the protein concentration of the secretome, it was possible to identify low-expression proteins, not detectable in plasma, with potential therapeutic implications [179].

Rocchiccioli et al. applied LC MS/MS to secretomes from fourteen carotid endarterectomy specimens, using their downstream distal side segments as controls, to obtain detailed protein profiles, thus allowing for the identification of 463 proteins, 31 of which were shown to be differentially secreted. Some of the latter were chosen for further validation by immunohistochemistry and their levels were assessed by ELISA on plasma samples from 34 patients and 10 healthy volunteers, confirming a significantly higher concentration of thrombospondin-1 and the vitamin D-binding protein in atherosclerotic subjects [21].

Another gel-free proteomic approach was adopted by Aragonès et al., which combined tandem immunoaffinity depletion, isobaric tags for relative and absolute quantification (iTRAQ) labeling, and nanoflow liquid chromatography coupled to high-resolution mass spectrometry for the analysis of secretomes from 12 carotid atherosclerotic plaque and 10 non-atherosclerotic mammary artery specimens [180]. By this approach, 162 proteins were quantified, including 25 proteins showing statistically significant differences. Validation by ELISA was performed for neutrophil defensin 1, apolipoprotein E, clusterin, and zinc-alpha-2-glycoprotein, which showed higher levels in secretomes from carotid atherosclerotic plaques. Although interesting, these results need to be validated on plasma from a large cohort of patients.

**Table 3:** Proteomics studies on human plaque secretomes. The sample source, the methodology applied, and the most relevant findings are reported. Reduced (↓) or increased (↑) levels of protein concentrations are indicated.

Samples	Proteomics	Main Findings	References
Secretome from normal carotid artery segments vs. non complicated plaque vs. complicated plaque with thrombus	2DE coupled with MALDI TOF MS	The more complicated the lesion, the higher the number of secreted proteins, suggesting the production of specific proteins relating to the complexity of the atherosclerotic lesion	[175]
Secretome from 35 atherosclerotic endarterectomies (10 femoral, 25 carotids) vs. 36 control endarteries (24 mammary, 12 radial)	2DE coupled with LC-MS/MS	Decreased secretion of HSP27 in complicated atherosclerotic plaques	[176]
21 carotid arteries secretomes—stenosing complicated zone (incubated in the presence or absence of atorvastatin) vs. the adjacent fibrous zone	2DE coupled with MALDI TOF MS/Q TOF MS	↑ 24 proteins ↓ 20 proteins; 66% of the proteins differentially released by atherosclerotic plaques reverted to control values after the administration of atorvastatin	[177]
Secretomes from 7 carotid arteries vs. 7 mammary arteries	SELDI TOF MS	↓ soluble tumor necrosis factor-like weak inducer of apoptosis (sTWEAK); sTWEAK concentrations negatively correlated with the carotid intima-media thickness (r 0.4; *p* 0.001)	[178]
Secretomes from three biological replicates of human atherosclerotic coronary arteries (APC), preatherosclerotic coronaries (PC) and mammaries (M)	1DE coupled with LC MS/MS	In total, 64 proteins were identified in the 3 replicates of at least one of the 3 groups and 15 secreted proteins have not been previously reported in plasma. Four proteins were significantly released in higher amounts by mammary tissue: gelsolin, vinculin, lamin A/C and phosphoglucomutase 5	[179]
Secretomes from carotid endarterectomy specimens of 14 patients	LC MS/MS	In total, 463 proteins were identified, of which 31 proteins were differentially secreted between plaques and the corresponding downstream segments	[21]
Secretomes from 12 carotid atherosclerotic plaque vs. 10 nonatherosclerotic mammary artery	Tandem immunoaffinity depletion, iTRAQ labeling, and nano LC-MS/MS	In total, 162 proteins were quantified, of which 25 were differentially expressed in secretome between carotid atherosclerotic plaque and non-diseased mammary artery. ↑ neutrophil defensin 1, apolipoprotein E, clusterin, and zinc-alpha-2-glycoprotein in CAP secretomes	[180]

### 3.3. Studies on Animal Models

Proteomics has been applied to widely used animal models of atherosclerosis to unveil significant insights into the molecular mechanisms influencing atherogenesis (Table 4). In this respect, the apolipoprotein E-deficient mouse is the most widely used murine model in cardiovascular research.

Mayr et al. analyzed aortic lesions from apolipoprotein E^-/-^ and wild type mice classified as light, medium, and severe according to lesion-covered areas on the aortic surface [29]. As expected, the authors found an increase in inflammatory cells, a decrease in VSMCs, and an accumulation of serum proteins associated with an impaired endothelial barrier function with lesion progression. Interestingly, immunoglobulins, which were barely detectable in apolipoprotein E^+/+^ mice, accumulated even in the aortas of young apolipoprotein E^-/-^ mice. The authors identified 79 differentially expressed spots. Moreover, they suggested an increase in oxidative stress together with lesion progression by evaluating the ratio between the oxidized and reduced forms of peroxiredoxin, the former resulting in a charge shift toward a more acidic isoelectric point. Overall, they found a linear relationship between the degree of peroxiredoxin-Cys oxidation and the extent of lesion formation in aortas of apolipoprotein E-deficient mice. A great deal of research has shown a contributory role of oxidative modifications (particularly LDL) within the arterial wall in the early events of atherogenesis [181,182]. The abovementioned results are in agreement with the evidence for a higher protein-sulfhydryl oxidation in unstable human carotid plaques, with respect to the stable ones, due in part to higher levels of sulfhydryl-thiolation, indicative of a more pronounced oxidative environment in advanced human carotid lesions [7,8].

Almofti et al. applied 2DE coupled to MALDI-TOF MS analysis to a rat model of atherosclerosis. They induced atherosclerosis by a single dose of vitamin D3 associated with a high-fat diet and identified 46 proteins differently expressed in diseased tissues. Among them, 18 proteins, including a group of oxidization-related enzymes, were found to be upregulated, while 28 proteins were found downregulated [183].

The vascular endothelium plays important physiological roles in vascular homeostasis, coagulation, inflammation, as well as in tissue growth and repair. The impairment of the endothelial function is an early event in atherosclerotic lesion formation leading to the overexpression of adhesion molecules, as well as the secretion of pro-inflammatory and chemotactic cytokines [184]. An affinity-based proteomic approach was used by Wu et al. [30] to identify vascular endothelial surface proteins differentially expressed in aortic tissues of apolipoprotein E-deficient mice. After in situ perfusion of the vascular bed with a solution containing a biotin derivative, biotinylated endothelial proteins were extracted, purified by affinity enrichment with streptavidin-agarose beads, and resolved by SDS-PAGE. The whole gel lanes were cut into slices and were subjected to tryptic digestion for nano-LC MS/MS analysis. In this way, 454 proteins, mainly extracellular or associated to the cell membrane, were identified. Among them, there were cell adhesion molecules, accounting for the largest category, followed by proteins involved in signal transduction and transport. Interestingly, proteins associated with immune and inflammatory responses were more than doubled in atherosclerotic aorta (13%) in comparison to normal aorta (6%). On the other hand, proteins involved in lipid metabolism were decreased by 34% in atherosclerotic aorta.

A rat model has been used in a proteomic study on the effects of blood shear stress on atherogenesis [185]. It is well known that blood shear stress affects endothelial cell shape and orientation, as well as vascular wall permeability. Indeed, regions of arterial branching or curvature, where blood flow is not uniform, are the preferential sites for lesion initiation. By comparing homogenates of aortas kept under two levels of shear stress in a perfusion culture system for 24 h, Qi et al. detected a reduced expression of protein Rho-GDP dissociation inhibitor alpha (Rho-GDIα) in low-shear stress conditions, and demonstrated, by siRNA technology, that this reduction enhances VSMC migration and apoptosis.

To identify early changes in protein expression during atherogenesis, Rodger et al. applied 2-DE coupled with MS to the analysis of the aortic arch of transgenic mice expressing a human apo(a) cDNA encoding seventeen KIV domains and human apo B100 [186]. By this approach, they identified a panel of differentially expressed proteins indicative of metabolic changes that precede the onset of atherosclerotic lesions.

Pelosi et al. applied a system approach by integrating information from histology indexes, as well as circulatory and local factors, to a high cholesterol-fed swine model to investigate the pathogenesis of coronary atherosclerotic lesions. This represents the model that best mimics human pathology. The main findings show that systemic inflammation is locally associated with macrophage/phagocytosis-related artery-specific protein expression, having an impact on atherosclerosis severity [36]. Rocchiccioli et al. obtained a map of 224 proteins secreted by coronary plaques in culture from the same pig model as Pelosi’s, and evidenced a strong relationship between VSMCs activation/migration and lesion development [37].

Hanzawa et al. performed proteomics on both the plasma and atherosclerotic tissue of apo E-deficient and wild type mice fed a high-fat diet, harvested at four different degrees of lesion development, evidencing quantitative variations of proteins involved in inflammation, thrombus formation, and vascular remodeling, according to the lesion progression [31].

Xu et al. applied an iTRAQ coupled with LC-MS/MS to measure protein-level changes in the ascending aortas of a cholesterol-fed rabbit model. By this approach, they identified 453 unique proteins, with 67 differentially expressed in response to hypercholesterolemia. Among them, there were apolipoproteins, extracellular matrix adhesion proteins, glycolytic enzymes, heat shock proteins, and proteins involved in immune defense, as well as a set of novel proteins not yet known for their involvement in atherogenesis that deserve further investigation [34].

ECM plays key roles in cell signaling and inflammatory processes during atherogenesis, as well as in stabilizing the fibrous cap during plaque development. However, the MS-based study on ECM is complicated by both the insolubility of its constituents and the presence of intracellular protein contaminants [187]. In this respect, Wierer et al. developed a very sensitive in-depth approach termed Quantitative Detergent Solubility Profiling (QDSP), which was applied to study the matrisome in intermediate and advanced lesions developed by apo E knock-out mice fed with a high-fat diet. Among all 5117 proteins identified, 182 showed differences concurrently with atherosclerotic plaque development. With regards to the insoluble ECM components, the expressions of 65 proteins, including collagens, matrix metalloproteinases, and macrophage-derived proteins, as well as proteins involved in vascular calcification, changed significantly [32].

Inflammation and proteolysis play central roles in atherosclerotic plaque degeneration towards instability, as also shown previously with regards to advanced human carotid lesions [5]. Vaisar et al. generated transgenic mice developing ruptured aortic plaques and assessed their suitability as a mouse model for studying plaque ulceration by comparing their proteomic profiles with those obtained from human carotid plaques [188]. In particular, the apo E^-/-^ mouse model overexpressed the urokinase-type plasminogen activator, a serine protease involved in tissue remodeling and cell migration. By shotgun proteomics, they evidenced common features leading to plaque instability, including extracellular matrix and basement-membrane proteolysis, suggesting that the loss of basement-membrane proteins may play a central role in advanced plaque rupture.

**Table 4:** Proteomics studies on animal models. The sample source, the methodology applied, and the most relevant findings are reported. Reduced (↓) levels of protein concentrations are indicated.

Samples	Proteomics	Main Findings	References
Aortas from 10-week-old, 12-month-old, and 18-month-old apolipoprotein E^-/-^ mice	2DE coupled with LC-MS/MS	In total, 79 protein species were identified as altered during various stages of atherogenesis. Immunoglobulin deposition, redox imbalance, and impaired energy metabolism preceded lesion formation in apolipoprotein E^-/-^ mice	[29]
Atherosclerotic aorta vs. normal aorta from Wistar rats (Atherosclerosis was induced by a single dose of vitamin D3 followed by a high fat diet)	2DE coupled with MALDI TOF MS	Found 46 differentially expressed proteins	[183]
Aorta from apo E^-/-^ mice vs. WT mice	Affinity proteomic strategy for in situ isolation and differential mapping of vascular endothelial proteins: perfusion with biotin, capturing with streptavidin chromatography, SDS-PAGE followed by LC-MS/MS	Here, 454 proteins identified, and 81 differentially expressed proteins involved in immune and inflammatory responses, cell adhesion, and lipid metabolism	[30]
Rat aorta cultured under two levels of shear stress, 5 and 15 dyn/cm^2^	2DE coupled with MALDI TOF MS	↓ Rho-GDIα in low shear stress vessels	[185]
Aortas from Lipoprotein(a) transgenic mice vs WT mice	2-DE followed by MALDI-TOF/TOF MS	Here, 34 differentially expressed proteins found involved in energy, redox, and lipid metabolism	[186]
Ascending arch of thoracic aorta from 24 male albino rabbits assigned randomly into 4 groups: control, cholesterol, cholesterol plus vitamin E and vitamin E groups	LC MS/MS	Differential expression of proteins following cholesterol and also vitamin E treatments	[33]
Secretomes of coronary arteries from pigs fed on standard or high-cholesterol diet	LC MS/MS	TNFα was identified as an associated plasma marker, oxLDL and HDL as relevant lipoproteins; macrophage function-related antioxidant Catalase enzyme, lysosome-associated Cathepsin D, S100-A10, and Transforming growth factor-beta-induced protein ig-h3 were identified and selected as associated to atherogenesis outcome	[36]
Low-dose streptozotocin-induced diabetic mouse model (10 animals)	LC MS/MS	Dysregulation of molecules involved in myogenesis, vascularization, hypertension, hypertrophy (associated with thickening of the aortic wall), and a substantial reduction in fatty acid storage. A novel finding is the pronounced downregulation of glycogen synthase kinase-3β (Gsk3β) and the upregulation of molecules linked to the tricarboxylic acid cycle (e.g., aspartate aminotransferase (Got2) and hydroxyacid-oxoacid transhydrogenase (Adhfe1)). In addition, pathways involving primary alcohols and amino acid breakdown are altered, potentially leading to ketone-body production. Conclusions: streptozotocin-induced diabetes mellitus in animals leads to a reduction in fatty acid biosynthesis and an upregulation of an alternative ketone-body formation pathway	[189]
Plasma and aortic tissue from apo E deficient and wild-type mice	ICAT followed by nanoLC-MS/MS	Here, 13 proteins in the plasma and 36 in the arterial tissues showed significant difference in abundance. These proteins were found to be components of inflammation, thrombus formation, and vascular remodeling, suggesting drastic and integrative alterations in accordance with atherosclerosis development	[31]
Cholesterol-fed rabbits	LC MS/MS iTRAQ	Here, 453 unique proteins were identified and quantified; 67 proteins differentially expressed, 62 higher (e.g., apolipoproteins, extracellular matrix adhesion proteins, glycolytic enzymes, heat shock proteins and proteins involved in immune defense) and 5 lower in ascending aortas from HCD-fed rabbits compared to controls	[34]
Secretomes of coronary arteries from pigs fed on standard or high-cholesterol diet	LC MS/MS	A wide coronary artery map of secreted proteins has been obtained in high-fat (HF) diet-induced ATS swine model and a significantly different expression of many proteins related to VSMC activation/migration has been identified	[37]
Aortas from apo E knock-out mice	LC MS/MS	Here, 5117 proteins were identified, 182 of which changed their expression status in response to vessel maturation and atherosclerotic plaque development. In the insoluble ECM proteome, 65 proteins significantly changed, including relevant collagens, matrix metalloproteinases and macrophage derived proteins	[32]
Aortas from SR-uPA^+/0^ (n = 6) vs. SR-uPA0/0 (n = 6) apo E^-/-^ mice; ruptured (n = 6) and stable (n = 6) areas of human carotid plaques	LC MS/MS	Here, 775 unique proteins were identified; overexpression of urokinase-type plasminogen activator may cause plaque rupture by activating proteolytic and proinflammatory mechanisms leading to ECM and basement-membrane protein depletion, as well as by upregulating the intracellular pathways related to cell–cell adhesion and cell–matrix adhesion. Several biochemical features in common between ruptured human carotid plaques and SR-uPA^+/0^ mouse aortas	[188]

## 4. Conclusions

In the last few years, proteomics has emerged as a powerful tool in the search for novel cardiovascular biomarkers with diagnostic/prognostic value, offering an unparalleled opportunity to capture the multifaceted aspects of atherosclerosis and its progression, and bridging the gap between fundamental research and practical clinical applications. Both lipoproteins and atherosclerotic tissues, particularly plaque extracts and secretomes, represent a rich source of information with regard to both early events of atherogenesis and predictors of acute clinical events. Furthermore, the analysis of LCM by proteomics, although in its infancy, could reveal valuable topological differences between specific areas of such a heterogeneous environment.

However, several issues hinder the step towards a translational medicine: in primis, the intrinsic complexity of the atherosclerotic tissue, its availability, as well as the choice of the proper controls, could hinder the interpretation of the results. Besides this, large-scale studies are required to validate the usefulness of the newly identified biomarkers. Finally, although several databases enclosing results from genome-wide association studies or gene expression profiling exist, as far as we know, no proteomics datasets focused on atherosclerosis are available. Therefore, in the near future, it will be essential to dedicate efforts to conducting a thorough investigation of the vast amount of data generated so far by proteomics, and to create shared datasets to collect all the identified markers. In this respect, artificial intelligence tools could represent a profound change in data analysis and interpretation, being able to perform both fast and iterative processing on a large amount of information, such as those obtained by means of all the abovementioned technologies.

## Figures and Tables

**Table 1:** Proteomic studies performed on purified lipoprotein fractions, investigating pathological conditions including coronary artery disease (CAD), acute myocardial infarction (AMI) (⁕), chronic kidney disease (CKD), end-stage renal disease (ESRD) (#), type 1 and 2 diabetes mellitus (T1DM and T2DM) (§), and experimental atherosclerosis (ǂ). Reduced (↓) or increased (↑) levels of protein concentrations are indicated.

	Lipoprotein Fractions	Purification	Proteomics	Main Findings	References
⁕	Group 1: HDL from 20 healthy volunteers; Group 2: HDL3 from 6 controls and 7 CAD patients; Group 3: HDL3 from 32 controls and 32 CAD; HDL from atherosclerotic tissue	Ultracentrifugation/immunoaffinity chromatography	LC-ESI-MS/MS analysis	48 proteins identified in HDL involved in lipid metabolism, complement regulation, proteinase inhibition, acute-phase response. Apo C-IV, paraoxonase-1, Complement C3, apo A-IV, apo E (confirmed by WB on HDL3 fraction from 64 subjects) enriched in HDL3 from CAD. More than 100 proteins identified in HDL from atherosclerotic plaque	[51]
⁕	Group 1: HDL3 from 6 CAD, 6 CAD + Statin/Niacin, 6 healthy controls; Group 2: HDL3 from 18 CAD, 18 CAD + Statin/Niacin. 12 months therapy	Sequential density ultracentrifugation	LC-FTICR MS	After 12 months of therapy, ↓ apo E, ↑ apo F and phospholipid transfer protein; combination therapy may revert CAD-associated changes in HDL3 protein composition	[77]
⁕	HDL from 7 hypercholesterolemia subjects and 9 normolipidemic subjects	Salt Gradient Ultracentrifugation	LC-MS/MS analysis	The quantitative HDL protein pattern correlates with the corresponding concentration and distribution of cholesterol from serum lipid measurements; hypercholesterolemia in unrelated individuals is the result of different deficiencies	[78]
⁕	HDL2 from 18 CAD and 20 controls	Sequential density ultracentrifugation	MALDI-TOF-TOF MS and pattern recognition analysis; LC-MALDI-TOF/TOF MS	HDL2 from CAD contains methionine sulfoxide residues in apolipoprotein A-I and elevated levels of apolipoprotein C-III; specific proteomic signatures of HDL2 accurately classify CAD and control subjects	[79]
⁕	HDL from 10 healthy men with low HDL cholesterol and 10 men with high HDL cholesterol levels challenged with endotoxin	Immunoaffinity chromatography (anti-apo A-I antibodies); density gradient ultracentrifugation	PS20 protein-chips (anti-apo A-I antibodies) coupled with SELDI-TOF MS analysis/2DE analysis	Profound changes in 21 markers (including SAA-1/2 and paraoxonase-1) were observed in the proteome in both groups; no relationship between baseline HDL cholesterol levels and HDL protein dynamics after endotoxin challenge	[80]
⁕	HDL from 39 new-onset AMI-patients vs 60 healthy individuals	Salt Gradient Ultracentrifugation	2DE coupled with MALDI-TOF MS analysis	Altered glycosylation pattern in AMI-patients within the first 6 h after the onset of the event	[81]
⁕	HDL from 10 healthy controls, 10 CAD and 10 ACS	Sequential ultracentrifugation	1DE coupled with LC-ESI-MS/MS analysis	67 HDL-associated proteins identified; ↓ apo A-IV, ↑ SAA, ↑ Complement C3 in ACS vs. both CAD and controls	[82]
⁕	HDL from CAD, ACS, and healthy controls	Sequential ultracentrifugation or gel filtration chromatography	LC-ESI-MS/MS analysis	↓ clusterin, ↑ apolipoprotein C-III in both CAD and ACS; HDL-proteome remodeling plays an important role for these altered functional properties of HDL (stimulation of proapoptotic pathways)	[83]
⁕	VLDL, LDL and HDL from four pooled plasma samples from 79 patients undergoing carotid endarterectomy and 57 healthy normolipidemic volunteers	Density gradient ultracentrifugation	2DE coupled with MALDI-TOF-MS	↑ acute-phase serum amyloid A protein (AP SAA) in all lipoprotein fractions, especially in LDL from atherosclerotic patients	[84]
⁕	HDL3 from 3 low HDL-c and 3 high HDL-c subjects	Density gradient ultracentrifugation	Top-Down Differential Mass Spectrometry: ETD LC-ESI-MS/MS	Proof-of-concept study; higher abundances of three apo C-III glycoforms in HDL3 from donors with low HDL-c	[85]
⁕	HDL from 10 congenital heart disease (CHD) patients vs. 10 controls	Sequential ultracentrifugation	iTRAQ technology coupled with nanoLC-MS/MS	196 proteins identified, 5 upregulated proteins (inflammatory reactions) and 7 downregulated (lipid metabolism) proteins; HDL proteome changes to a pro-atherogenic profile in CHD patients	[86]
⁕	HDL_2_ and HDL_3_ from 40 ACS vs. 40 controls	Two-step discontinuous density-gradient ultracentrifugation	2DE and 2DIGE analysis coupled with MALDI-TOF/TOF MS	17 differentially expressed HDL-associated proteins identified, shifting to a dysfunctional HDL subfractions	[87]
⁕	HDL from diabetes and CVD	Sequential ultracentrifugation	MAP/LC-MRM/MS	69 and 32 HDL proteins quantified, respectively, with multi-analyte profiling (MAP) and multiple reaction monitoring (MRM); high-throughput approach to examine changes in HDL proteins in diabetes and CVD	[88]
⁕	HDL from 21 CAD pre- and post- percutaneous transluminal coronary angioplasty (PTCA)	Immunoaffinity chromatography (anti-apo A-I antibodies)	Quantitative ^16^O/^18^O analysis/iTRAQ technology coupled with LC–MS/MS/system biology analysis	225 identified proteins; high protein variability in HDL composition between individuals; post-PTCA increase in two protein clusters that included several apolipoproteins, fibrinogen-like protein 1 and other intracellular proteins, and a decrease in antithrombin-III, annexin A1 and several immunoglobulins	[89]
⁕	HDL from 20 subjects at risk for CAD: 10 patients had CAD and 10 did not	Sequential ultracentrifugation	Shotgun proteomic, glycomic, and ganglioside analyses using LC-MS	Combined HDL proteomic and glycomic profiles distinguished at-risk subjects with atherosclerosis from those without; CAD patients had ↓ apo A-I, ↓ apo A-II, ↓ apo E, ↓ SAA2, ↓ SAA4 (*p* = 0.007), ↑ sialylated glycans	[90]
⁕	HDL from 33 hypercholesterolemic subjects included in a clinical trial evaluating effects of virgin olive oil (VOO) and phenol-enriched VOO	Sequential density ultracentrifugation	Nano LC-MALDI MS/MS and nano LC-ORBITRAP-ESI MS/MS	127 HDL-associated proteins identified; 15 differentially expressed proteins involved in liver X receptor-retinoid X receptor activation, acute phase response, and atherosclerosis; consumption of VOO or phenol-enriched VOOs, has an impact on the HDL proteome in a cardioprotective mode by upregulating proteins related to cholesterol homeostasis, protection against oxidation and blood coagulation while downregulating proteins implicated in acute-phase response, lipid transport, and immune response	[91]
⁕	HDL from 54 systemic Lupus Erythematosus (SLE) patients (with and without atherosclerotic plaque), 25 controls, patients with type 1 diabetes with or without coronary artery calcification	Sequential density gradient ultracentrifugation	Targeted proteomics (18 proteins): LC-ESI-MS/MS analysis	↓ PON3 in HDL of both SLE and T1DM patients with subclinical atherosclerosis	[92]
⁕	LDL and HDL from 10 healthy individuals with normal LDL-C treated with rosuvastatin for 28 days	Size-exclusion chromatography	LC-ESI-MS/MS analysis	Among the differentially expressed proteins, α-1-antirypsin increased dramatically in HDL; enhanced HDL anti-inflammatory activity, contributing to the non-lipid lowering beneficial effects of statins	[93]
⁕	HDL from 5 chronic heart failure (CHF) patients vs. 5 controls	Sequential ultracentrifugation	SCX/RP LC–MS/MS	494 proteins identified; 23 newly identified HDL-associated proteins; 223 bacterial peptides were found in both CHF and controls	[94]
⁕	HDL from 14 abdominal aortic aneurism (AAA) patients vs. 7 controls	Sequential ultracentrifugation	iTRAQ technology coupled with nanoLC-MS/MS	↑ peroxiredoxin-6, ↑ class I histocompatibility antigen, ↑ retinol-binding protein 4, ↑ paraoxonase/arylesterase 1, ↓ α-2 macroglobulin, ↓ C4b-binding protein	[95]
⁕	HDL from 10 CVD vs. 7 controls	Two-step discontinuous density-gradient ultracentrifugation	nLC-MS/MS analysis	118 proteins identified; 10 proteins positively associated with the combined level of persistent organic pollutants (POPs) or with highly chlorinated polychlorinated biphenyls (PCB) congeners. Among these, cholesteryl ester transfer protein and phospholipid transfer protein, as well as the inflammatory marker serum amyloid A, were found. The serum paraoxonase/arylesterase 1 activity was inversely associated with POPs. Pathway analysis demonstrated that upregulated proteins were associated with biological processes involving lipoprotein metabolism, while downregulated proteins were associated with processes such as negative regulation of proteinases, acute phase response, platelet degranulation, and complement activation	[96]
⁕	HDL from 126 subjects with clinical indication for a coronary computed tomography angiography (CCTA)	High-resolution size exclusion chromatography followed by phospholipid-associated proteins capture (Calcium Silicate Hydrate)	LC-ESI-MS/MS	72 HDL-associated proteins detected in at least 75% of subjects; 13 proteins significantly associated with calcified plaque burden including cathelicidin antimicrobial peptide, gelsolin (GELS), kininogen-1 (KNG1), and paraoxonase-1 (inverse relationships), apo A-IV, vitamin D binding protein, alpha-2- macroglobulin, and apo C-II (positive relationships); 15 proteins significantly associated with non-calcified plaque burden including apo A-I, apo F, antithrombin III, and apo C-I (inverse relationships), serum amyloid A1, immunoglobulin heavy constant alpha 1, complement factor B, complement C2, complement C3, complement bC1s subcomponent (positive relationships); among the evaluated risk factors, BMI has the greatest overall impact on the protein composition of HDL	[97]
⁕	HDL from heart failure patients: cardiovascular deaths vs. survivors (1:1)	Calcium silicate matrix	nLC-MS/MS analysis	647 proteins identified; 49 HDL proteins were significantly different; a set of 12 selected proteins predicted death with 76% accuracy	[98]
⁕	HDL from 943 participants without prevalent myocardial infarction referred for coronary angiography in the CASABLANCA study	^15^NHis_6_Apo A-I was added to human serum, incubated, diluted, and then purified using PhyTips (Phynexus, San Jose, CA, USA), packed with Ni-NTA HisBind Superflow stationary phase	Targeted proteomics (apolipoprotein A-I, C-1, C-2, C-3, and C-4): LC-ESI-MS/MS analysis	An HDL apolipoproteomic score is associated with the presence of CAD, independent of circulating apo A-I and apo B concentrations and other conventional cardiovascular risk factors. Among individuals with CAD, this score may be independently associated with cardiovascular death	[99]
⁕	Apolipoprotein AI-associated lipoproteins from 231 healthy individuals and patients with CAD	Metal chelate affinity chromatography	Targeted proteomics (21 proteins): LC-MS/MS analysis	A multiplexed proteomic assay useful for the estimation of cholesterol efflux and CAD risk in the clinical laboratory	[100]
⁕	HDL from 8 patients with complete deficiency of cholesteryl ester transfer protein (CETP) vs. 8 normolipidemic healthy subjects	Sequential ultracentrifugation	LC-MS/MS analysis	79 HDL-associated proteins identified, involved in lipid metabolism, protease inhibition, complement regulation, and acute-phase response, including 5 potential newly identified HDL-associated proteins; ↑ apo E, ↑ Complement C3, C4a, C4b, and C9, ↑ apo C-III	[101]
⁕	HDL from patients after acute ischemic stroke at 2 time points (24 h, 35 patients; 96 h, 20 patients) and from 35 control subjects	Sequential density ultracentrifugation	Data-dependent acquisition (DDA) mass spectrometry and parallel reaction monitoring (PRM) mass spectrometry	Some proteins involved in acute phase response and platelet activation were significantly altered in stroke HDL at 24 and 96 h (*p* < 0.05). Accordingly, cholesterol efflux capacity was reduced by 32% (*p* < 0.001) at both time points	[102]
⁕	HDL from 46 incident new CVD and 46 one-to-one matched controls, at various stages of CKD	Sequential ultracentrifugation	Targeted proteomics (31 proteins): LC-MS/MS analysis	PON1, PON3, LCAT, and apolipoprotein A-IV levels inversely associated with incident CVD events in CKD patients	[103]
⁕	HDL and LDL from pooled plasma samples from 75 patients undergoing carotid endarterectomy and 50 healthy normolipidemic volunteers	Density gradient ultracentrifugation	nanoLC-MS/MS	Protein signatures specific for patients with “hard” or “soft” carotid plaques	[56]
#	HDL from 27 ESRD vs. 19 healthy subjects	Salt Gradient Ultracentrifugation	LC-MS/MS Analysis	35 HDL-associated proteins identified; antitrypsin, retinol-binding protein 4 (RBP4), transthyretin, apo A-VI, and further minor proteins were exclusively detected in uremic HDL; ↑ SAA1, ↑albumin, ↑lipoprotein-associated phospholipase A2, ↑ apo C-III, ↓ apo A-I, ↓ apo A-II	[104]
#	HDL from 7 chronic hemodialysis patients vs. 7 healthy controls	Sequential density ultracentrifugation	iTRAQ technology coupled with 2D nano-LC-MALDI-TOF/TOF MS	122 proteins identified, 40 proteins differentially expressed	[105]
#	HDL from 34 ESRD vs. 17 healthy controls	Sequential density ultracentrifugation	HPLC coupled with MALDI-TOF-TOF MS	SAA is enriched in HDL from ESRD patients correlating with its reduced anti-inflammatory capacity	[106]
#	HDL from 10 ESRD vs. 10 healthy controls	Sequential ultracentrifugation	1DE coupled with LC-ESI-MS/MS analysis	49 HDL-associated proteins identified; ↑ surfactant protein B (SP-B), ↑ apolipoprotein C-II, ↑ serum amyloid A (SAA), ↑ α-1-microglobulin/bikunin precursor; SAA promotes inflammatory cytokine production	[107]
#	HDL from 40 ESRD vs. 20 controls	Sequential ultracentrifugation	LC-ESI-MS/MS analysis	63 identified proteins; ↑ 22 proteins, ↓ 6 proteins; HDL proteome is extensively remodeled in uremic subjects	[108]
#	HDL from 509 CKD; estimated glomerular filtration rate (eGFR) > 60 mL/min/1.73 m^2^ vs. eGFR < 15 mL/min/1.73 m^2^	Two-step density gradient ultracentrifugation	nLC-MS/MS analysis (targeted proteomics: quantification of 38 HDL-proteins)	↑ retinol binding protein 4, ↑ apo C-III, ↑ apo L1, ↓ vitronectin	[109]
#	HDL from 143 patients starting hemodialysis vs. 110 patients with advanced CKD	Two-step density gradient ultracentrifugation	nLC-MS/MS analysis (targeted proteomics: quantification of 38 HDL-proteins)	↑ serum amyloid A1, A2, and A4, ↑ hemoglobin-b, ↑ haptoglobin-related protein, ↑ cholesteryl ester transfer protein, ↑ phospholipid transfer protein, ↑ apo E; hemodialysis initiation is associated with higher concentrations of HDL-proteins related to inflammation and lipid metabolism	[110]
#	HDL from 9 non-diabetic hemodialysis patients vs. 8 control patients	Sequential ultracentrifugation	nLC-Quadrupole-Orbitrap-MS	326 proteins identified; ↑ 10 proteins (UDP-glucose: glycoprotein glucosyltransferase 1, Beta-2-microglobulin, Pulmonary surfactant-associated protein B, Protein AMBP, Insulin-like growth factor II, Immunoglobulin heavy constant alpha 2, Immunoglobulin lambda constant 2, HLA class I histocompatibility antigen B-58 alpha chain, Complement factor D, Inter-alpha-trypsin inhibitor heavy chain H1), ↓ 9 proteins (Guanylin, Calpain-1 catalytic subunit, Keratin, type I cytoskeletal, Ras-related protein Rab-6B, Ganglioside GM2 activator, Prostaglandin-H2 D-isomerase, Secretoglobin family 3A member 2, Thioredoxin-dependent peroxide reductase mitochondrial, Solute carrier family 2 facilitated glucose transporter member 2) involved in lipid metabolism, hemostasis, wound healing, oxidative stress, and apoptosis pathways	[111]
§	HDL from 30 T1DM vs. 30 controls	Single Vertical Spin Density Gradient Ultracentrifugation	LC-ESI-MS/MS analysis	Compromised HDL anti-oxidant functions due to higher abundance of irreversible PTMs of proteins in T1DM	[112]
§	HDL from 191 T1DM subjects	Sequential ultracentrifugation	Targeted proteomics (46 proteins): LC-ESI-MS/MS analysis	8 proteins associated with albuminuria including AMBP (α1-microglobulin/bikunin precursor) and PTGDS (prostaglandin-H2 D-isomerase) that strongly and positively associated with the albumin excretion rate. PON1 and PON3 levels in HDL strongly and negatively associated with the presence of coronary artery calcium. Only PON1, associated with both albumin excretion rate and coronary artery calcification. The HDL proteome is remodeled in type 1 diabetes mellitus subjects with albuminuria	[113]
§	HDL from 26 patients with T1DM vs. 13 healthy controls	High-resolution size exclusion chromatography followed by phospholipid-associated proteins capture (Calcium Silicate Hydrate)	nLC-TripleTOF-MS	78 HDL-bound proteins were measured; youths with T1DM show proteomic alterations of their HDL compared to healthy controls, despite similar concentration of HDL cholesterol. The influences of these compositional changes on HDL function are not yet known	[114]
§	HDL from two cohorts of T1DM patients (n = 47, n = 100). T1DM without complications vs. T1DM with complications	Sequential density ultracentrifugation	Targeted proteomics (35 proteins): LC-MS/MS analysis	Elevated concentrations of M-HDL particles and elevated levels of HDL-associated PON1 may contribute to long-term protection from the vascular complications of diabetes by pathways that are independent of total cholesterol and HDL cholesterol. ↑ apo C-I, apo E, complement C3, apo C-II, apo M, and PON1, ↓ apo A-IV and retinol-binding protein 4 (RBP4). In the female subgroup analysis: ↑ apo C-I and PON1, ↓ apo A-IV. Targeted proteomics in the replication cohort: ↑ PON1	[115]
§	HDL from 9 newly diagnosed T2DM patients and 8 healthy controls	Immunoaffinity chromatography	LC-MS/MS	Plasma adiponectin levels were reduced in subjects with T2DM, which was directly associated with suppressed ABCA1-dependent cholesterol efflux capacity of HDL. The fractional catabolic rates of HDL cholesterol, apo A-II, apo J, apo A-IV, transthyretin, complement C3, and vitamin D-binding protein (all *p* < 0.05) were increased in subjects with T2D. Despite the increased HDL flux of acute-phase HDL proteins, there was no change in the proinflammatory index of HDL. Although lecithin-cholesterol acyl transferase (LCAT) and CETP activities were not affected in subjects with T2DM, LCAT was inversely associated with blood glucose and CETP was inversely associated with plasma adiponectin. The degradation rates of apo A-II and apo A-IV were correlated with hemoglobin A1c. In conclusion, there were in vivo impairments in HDL proteome dynamics and HDL metabolism in diet-controlled patients with T2DM	[116]
§	HDL from obese adolescents with T1DM (25 treated with metformin as adjunct therapy and 10 with placebo): double-blind, placebo-controlled clinical trial	Size-exclusion chromatography followed by lipophilic absorption	Data-independent acquisition (DIA) mass spectrometry	Two proteins out of eighty-four were significantly affected by metformin treatment, peptidoglycan recognition protein 2 and alpha-2-macroglobulin. Metformin did not significantly affect cholesterol efflux capacity (CEC). Changes in affected HDL proteins did not correlate with CEC	[117]
§	HDL2 and HDL3 from case–control study that enrolled 50 T1DM and 30 non-diabetes control individuals	Discontinuous density ultracentrifugation	45 proteins quantified by parallel reaction monitoring	13 proteins in HDL2 and 33 in HDL3 were differentially expressed in T1DM. Six proteins related to lipid metabolism, one to inflammatory acute phase, one to complement system and one to antioxidant response were more abundant in HDL2, while fourteen lipid metabolism, three acute-phase, three antioxidants and one transport were found in HDL3 of T1DM subjects. Three proteins (lipid metabolism, transport, and unknown function) were more abundant in HDL2, and ten (lipid metabolism, transport, protease inhibition) were more abundant in HDL3 of controls	[118]
ǂ	HDL from C57BL/6 mice challenged with intraperitoneal endotoxin vs. controls injected with saline	Sequential ultracentrifugation	Two-dimensional gel electrophoresis (2DE) coupled with LC-MS/MS analysis	↑ SAA, apo E, apo A-IV, apo A-V; ↓ apo A-I, apo A-II	[119]
ǂ	HDL from C57BL/6j mice following 24 weeks on saturated fatty acids (SFA)- or monounsaturated fatty acids (MUFA)-enriched high-fat diets (HFDs) or low-fat diet	Fast protein liquid chromatography (FPLC)	nLC-ESI-MS/MS analysis	HDL particles were enriched with acute-phase proteins (serum amyloid A, haptoglobin, and hemopexin) and depleted in paraoxonase-1 after SFA-HFD in comparison with MUFA-HFD	[120]
ǂ	HDL from knock-out (for apo A-I, apo A-II or apo A-IV) and wild type C57BL/6J mice	High-resolution size exclusion chromatography followed by phospholipid-associated proteins capture (Calcium Silicate Hydrate)	LC-ESI-MS/MS analysis	The loss of apo A-I or apo A-II massively reduced HDL lipids and changed the plasma size pattern and/or abundance of several plasma proteins. Surprisingly though, many HDL proteins were not affected, suggesting they assemble on lipid particles in the absence of apo A-I or apo A-II. In contrast, apo A-IV ablation had minor effects on plasma lipids and proteins, suggesting that it forms particles that largely exclude other apolipoproteins. Overall, the data indicate that distinct HDL subpopulations exist, and that they do not contain, nor depend on, apo A-I, apo A-II or apo A-IV, and these contribute substantially to the proteomic diversity of HDL	[93]
ǂ	HDL from Scavenger receptor class B type I^−/−^ mice vs. wildtype (WT) mice	Apo B containing lipoproteins precipitation followed by ultracentrifugation	LC–MS/MS analysis/2DE analysis	↓ apo A-I, ↓ PON1, ↑ SAA, ↑ apo A-IV, ↑ α-1-antitrypsin; impaired cholesterol homeostasis in macrophages, and reduced anti-oxidative and anti-inflammatory effects	[121]

**Table 2:** Proteomics studies on human plaque extracts. The sample source, the methodology applied, and the most relevant findings are reported. Reduced (↓) or increased (↑) levels of protein concentrations are indicated.

Samples	Proteomics	Main Findings	References
10 coronary arteries from CAD vs. 7 coronary arteries from normal individuals	2DE coupled with LC-MS/MS	Increased expression of the ferritin light chain in CAD (1.41 vs. 0.75; *p* = 0.01)	[156]
5 stable carotid plaques vs. 6 lesions with a thrombus	2DE coupled with MALDI-TOF/TOF MS	Expression of six isoforms of alpha1 anti-trypsin (AAT) in advanced plaques, one of which was uniquely expressed in thrombus-containing plaques	[157]
7 atherosclerotic aortas vs. 7 normal aortas specimens from the same patients	2DE coupled with MALDI TOF MS	27 differentially expressed proteins identified involved in a number of biological processes, including calcium-mediated processes, migration of vascular smooth muscle cells, matrix metalloproteinase activation and regulation of pro-inflammatory cytokines	[158]
35 human coronary atherosclerotic specimens (frozen, embedded, LCM specimens)	LC-MS/MS	806 proteins identified; first large-scale proteomics map of human coronary atherosclerotic plaques	[159]
10 carotid plaques	2DE	Synthetic gel from 2DE	[160]
29 unstable carotid plaques vs. 19 stable plaques	2DE coupled with MALDI TOF MS	57 spots identified ↓ SOD3, GST, HSP27, HSP20, annexin A10, and Rho GDI ↑ ferritin light subunit, SOD2 and fibrinogen fragment D	[6]
10 complicated segments in the internal carotid artery vs. more stable segments in the common carotid artery of the same patients	2-D DIGE coupled with nano-LC ESI MS/MS	↑ Protein S100A10, Fibrinogen beta, Ferritin light chain, AAT, Protein S100-A9, Serum albumin ↓ Apolipoprotein E, Actin, cytoplasmic 1, L-Lactate dehydrogenase B, Myosin regulatory light polypeptide 9, Acetyl-CoA acetyltransferase, mitochondrial, Actin, aortic smooth muscle, Transgelin, Calponin-1, Tropomyosin beta, Annexin A5, Myosin light polypeptide 6	[161]
80 carotid plaques from patients who had a cardiovascular event vs. 80 carotid plaques from patients who remained stable during follow-up	Two-dimensional LC-MS/MS	Plaque osteopontin levels in single lesions are predictive for cardiovascular events in other vascular territories. Local atherosclerotic plaques are a source of prognostic biomarkers with a high predictive value for secondary manifestations of atherosclerotic disease	[162]
Intimal proteome from the human atherosclerotic coronary artery (LCM) vs. preatherosclerotic coronary vs. radial arteries	2-D DIGE coupled with MALDI TOF/TOF MS	13 proteins were differentially expressed (7 upregulated and 6 downregulated), and are implicated in the migrative capacity of vascular smooth muscle cells, extracellular matrix composition, coagulation, apoptosis, heat shock response, and intraplaque hemorrhage deposition	[163]
Noncomplicated and complicated plaques from carotid atherosclerotic lesions	Enrichment step with Proteominer. 1DE and 2DE coupled with nLC-MS/MS	Novel low-abundance proteins were associated very well with biological alterations related to atherosclerosis	[164]
38 carotid atherosclerotic plaques pooled into two samples and analyzed in triplicate	Multidimensional LC-MS/MS	A total of 4702 proteins were identified from atherosclerotic plaques at a false discovery rate (FDR) of 1%, of which 3846 were identified with at least 2 unique peptides	[165]
Fibrotic and hemorrhagic carotid atherosclerotic plaques from coronary patients	Enrichment step with Proteominer. 2DE coupled with nLC-MS/MS	118 proteins, differentially expressed in fibrotic and hemorrhagic plaques, allowed for the identification of three biological processes related to atherosclerosis (platelet degranulation, vascular autophagy and negative regulation of fibrinolysis). Combinations of such circulating biomarkers could be used to stratify coronary patients	[166]
Human coronary thrombus	2-DE followed by MALDI MS/MS 1-DE followed by LC–MALDI-MS/MS 1-DE followed by LC–ESI-MS/MS)	708 proteins were identified in the thrombus. A positive correlation of 5 proteins (fermitin homolog 3, thrombospondin-1, myosin-9, beta parvin and ras-related protein Rap-1b) with CD41 was found, pointing out the potential activation of a focal adhesion pathway within thrombus platelets	[167]
Different regions of human carotid plaques from men and women	2-DE followed by MALDI-TOF MS/nLC-MS/MS	Expression levels of 18 proteins were significantly altered in plaque regions compared to the internal control region. In total, 9 proteins showed site-specific alterations, irrespective of gender, with clear associations to extracellular matrix remodeling; 5 proteins displayed gender-specific alterations with 2-DE, with two alterations validated by nLC-MS/MS. Gender differences in ferritin light chain and transthyretin were validated using both techniques. The validation of immunohistochemistry confirmed significantly higher levels of ferritin in plaques from male patients	[168]
23 lipid-rich plaques and 13 non-lipid-rich plaques	LC MS/MS	Library of 423 proteins that are present in coronary plaque debris; MMP-9 and another 5 plaque-enriched proteins (lipopolysaccharide binding protein, Annexin A5, eukaryotic translocation initiation factor, syntaxin 11, cytochrome B5 reductase 3) significantly enriched in plaque and in plasma after plaque disruption	[169]
28 vulnerable carotid plaques vs. 30 stable plaques	iTRAQ nanoLC MS/MS	28 proteins were differentially expressed, including alpha-2-macroglobulin (a2M) and heparin cofactor II (HCII). The plasma level of a2M was found to be positively correlated, while HCII level was negatively correlated, with the higher vulnerability of carotid plaques. Both proteins were efficient in differentiating stable and vulnerable carotid plaques	[170]
Human carotid endarterectomy specimens from 6 symptomatic versus 6 asymptomatic patients	(Three steps extraction of carotid plaque ECM) nanoLC MS/MS	A 4-biomarker signature (matrix metalloproteinase 9, S100A8/S100A9, cathepsin D, and galectin-3-binding protein) improved risk prediction and was successfully replicated in an independent cohort in the SAPHIR study	[171]
Human coronary arteries (n = 100) and aortas (n = 100)	LC MS/MS	Identified hundreds of proteins (n = 1925) and numerous networks and pathways that are associated with early atherosclerosis	[11]
Carotid plaques from 10 men vs. 10 women	LC MS/MS	Sex-specific differences were identified both in general levels and from the lesion region-specific perspective. In this study, men were shown to have greater levels of inflammatory response proteins like lysozyme C and sPLA2, and women to have greater levels of serine protease inhibitors and afamin. These differences in the proteome may be suggestive of women developing plaques with a lower inflammatory profile, and greater stability, than men	[172]
Coronary arteries at different stages of development, from 15 patients	2-DE followed by MALDI-TOF MS	The amounts of the following proteins were increased in stable atherosclerotic plaques at the stage of lipidosis and fibrosis: vimentin, tropomyosin β-chain, actin, keratin, tubulin β-chain, microfibril-associated glycoprotein 4, serum amyloid P-component, and annexin 5. In plaques at the stage of fibrosis and calcification, the amounts of mimecan and fibrinogen were increased. In the unstable atherosclerotic plaque of the necrotic–dystrophic type, the amounts of human serum albumin, mimecan, fibrinogen, serum amyloid P-component and annexin were increased	[173]

## Data Availability

No new data were created or analyzed in this study. Data sharing is not applicable to this article.
